# Application of computer-based testing in the Korean Medical Licensing Examination, the emergence of the metaverse in medical education, journal metrics and statistics, and appreciation to reviewers and volunteers

**DOI:** 10.3352/jeehp.2022.19.2

**Published:** 2022-01-13

**Authors:** Sun Huh

**Affiliations:** Department of Parasitology and Institute of Medical Education, College of Medicine, Hallym University, Chuncheon, Korea; The Catholic University of Korea, Korea

## Computer-based testing adopted for the Korean Medical Licensing Examination on January 6–7, 2022

**Fig. 1.**
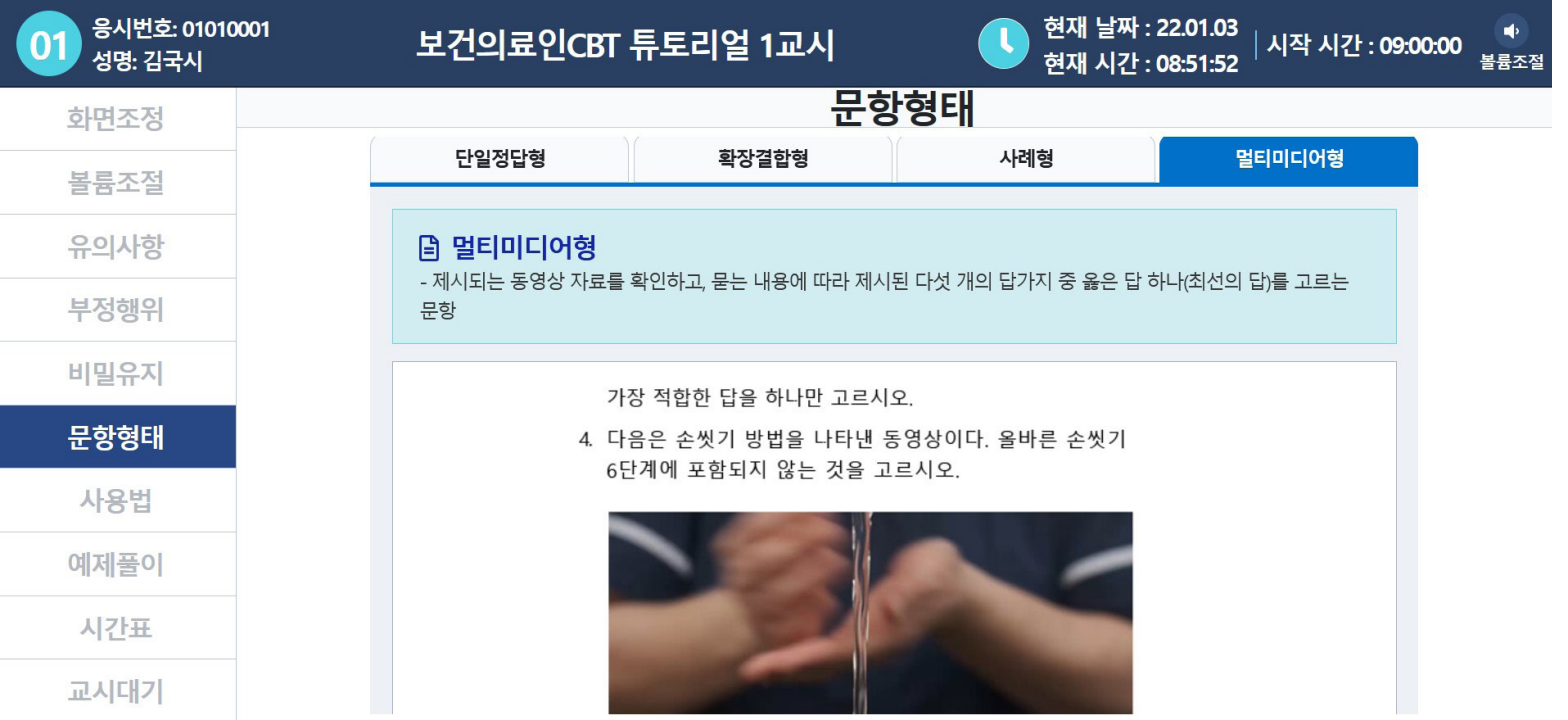
Screenshot of the mock test of computerized adaptive testing of the Korean Medical Licensing Examination in 2021, provided by the Korea Health Personnel Licensing Examination Institute.

On January 6–7, 2022, computer-based testing (CBT) was adopted for the Korean Medical Licensing Examination (KMLE) [1] (Fig. 1). This is the first instance of CBT adoption for any of the 26 health professional licensing examinations in Korea. Of course, smart device-based testing (SBT), a variant of CBT, was already adopted for the Korea Emergency Medicine Technician Licensing Examination in 2017 [2]. Examinees’ demographic characteristics and perceived acceptability of SBT did not affect the SBT mock test scores of emergency medicine technician students in Korea [3]. After implementing CBT in the KMLE, it will be expanded to all other 24 professions, including dentists, oriental medicine doctors, and care workers, by 2025 [1].

On March 21, 2003, presenters first proposed CBT and computerized adaptive testing (CAT) for the KMLE at a seminar held by the Institute of Medical Education of Hallym University at the Chuncheon Sejong Hotel. At that time, the staff members of the Korea Health Personnel Licensing Examination Institute (KHPLEI) attended the seminar. Thus, 19 years have passed since CBT and CAT were suggested for licensing examinations in Korea.

In November 2011, the Standing Committee of the KMLE recommended introducing computerized testing to the KMLE, including CBT and CAT [4]. I also suggested improving the quality of the KMLE through the introduction of computerized testing and recommended that medical schools should prepare for the new testing environment of the KMLE [4]. The 6th president of the KHPLEI, Dr. Myung-Hyun Chung (August 1, 2012–July 31, 2015), announced that research and development on CBT and ubiquitous-based testing would be accelerated in January 2013 [5]. Research on CBT then became more active. For example, the linear programming method was suggested to construct equated-item sets that reflect each content area. The best choice was to divide the predicted correct answer rate into 2 or 3 difficulty boundaries regardless of common items [6]. Because SBT was already well implemented in the Korea Emergency Medicine Technician Licensing Examination [3], there was no need to repeat an evaluation of the examinees’ adaptability to CBT. The only step left was decision-making by the president of KHPLEI. Dr. Yoon-Seong Lee, the 8th president, finally decided to introduce CBT.

CBT has already been a very common testing platform at most medical schools. For example, at Hallym University, CBT was introduced in 1999 for clinical course testing. In my parasitology class, CBT was already introduced in 1993. My students have had no difficulties in taking CBT. A new task after adopting CBT is to guarantee item quality, including that of audio-visual materials. CBT items should be more clinically oriented than paper-and-pencil materials to test examinees’ clinical competency. Furthermore, the number of items to measure the examinee’s latent traits should be re-considered. A correct distribution of core clinical content is required, and the balance between reliability and core clinical presentations should be guaranteed to ensure desirable item numbers. The standard setting for CBT, including the modified Angoff, modified Ebel, and Hofstee methods, should be implemented soon, instead of using a cut score to ensure a success rate of 60% [7,8]. It is necessary to maintain a consistent passing rate regardless of item difficulties.

## Study size estimation and arrival of the metaverse in medical training in Korea

Two invited reviews were published last year: “Sample size determination and power analysis using the G*Power software” by Kang [9] and “Educational applications of metaverse: possibilities and limitations” by Kye et al. [10]. There was also an invited editorial: “Training in lung cancer surgery through the metaverse, including extended reality (XR), in the smart operating room of Seoul National University Bundang Hospital, Korea” by Koo [11].

Although Dr. Kang is an anesthesiologist, he studied statistics after graduating from Korea National Open University with a major in Statistics. I invited him to serve as a statistical editor in 2021. I asked him to submit a review on determining the study size for authors and reviewers of the journal. When I read manuscripts submitted to the journal, the most challenging task is to check the appropriateness of study size estimation, especially since many manuscripts do not describe it. If estimation before the study was not possible, it is recommended to conduct a post-hoc analysis. Dr. Kang stated that “the null and alternative hypothesis, effect size, power, alpha, type I error, and type II error should be described when calculating the sample size or power” [9]. I hope that this invited methodological review will be helpful for authors and researchers.

The year 2021 was the year of the metaverse [12]. Although the term “metaverse” was known beforehand, and it had already been developed as a technology, its use exploded in the coronavirus disease 2019 (COVID-19) pandemic era since many people could not meet face to face. This also occurred in health education. My university also trained faculty members on how to use Gather, one of the virtual educational spaces of metaverse platforms, called the virtual world. Unfortunately, I could not apply this platform for my medical students in 2021 due to my unpreparedness. I will use this platform or another virtual educational space for this year’s parasitology class. Several types of the metaverse, including augmented reality (AR), mirror world, lifelogging, and virtual reality (VR), have already been introduced in medical and health education.

**Fig. 2.**
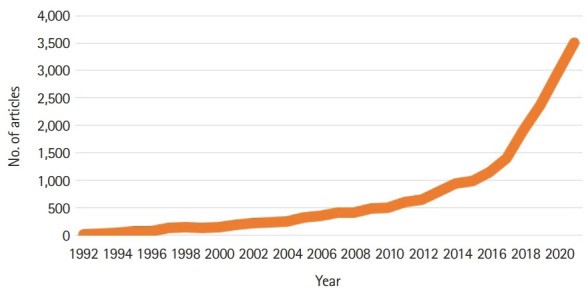
Number of articles on the 4 types of the metaverse in PubMed by year with the search term “(((augmented reality) OR (lifelogging)) OR (mirror world)) OR (virtual reality)” [cited 2022 Jan 8].

I did a PubMed search with the keyword “metaverse,” and only 11 articles were found on January 8, 2022. However, this does not mean that the number of articles on metaverse is small. Before that, articles on the 4 types of the metaverse have been published frequently. These publications soared in 2021 (Fig. 2, Supplement 1) and the term “metaverse” began to appear in biomedical articles in 2020 and 2021. There are already 4 articles on this topic in the *Journal of Educational Evaluation for Health Professions* (JEEHP): VR endotracheal intubation training [13]; digital technologies introduced in medical and dental education [14]; a simulation-based blended training model for nurses [15], and VR training to decreases rates of needle stick/sharp injuries [16].

According to the usage trends of the term “metaverse,” a review article was invited as an abridged English translation of the issue report by the Korea Education and Research Information Service [10]. Dr. Bokyung Kye, Director of the Global Policy Research Section, kindly provided this report for the journal’s readers. This review article is a broad introduction to the educational application of the metaverse. The definition of the relevant terminology was explained, and current examples of metaverse use were introduced. It also included applications in health education. I hope that this accessible and comprehensive review article will be helpful for medical and health educators to understand the concept of the metaverse and the strengths and limitations of its application. Furthermore, Koo [11], a senior reporter from JTBC, one of the well-known broadcasting companies in Korea, wrote an editorial on the use of the metaverse to train chest surgeons at Seoul National University Bundang Hospital. This training course was an example of XR implemented by mixing AR and VR. XR refers to all VR technologies ranging from VR to mixed reality and AR.

## Journal metrics and statistics

In 2021, JEEHP received its first Journal Citation Indicator (JCI) value, 0.51, in June 2021. This score means that the citation impact of articles from 2017 to 2019 was about half of the average citation impact of Web of Science Core Collection journals. JEEHP’s JCI ranking in the scientific education category was 9th out of 35 Emerging Sources Citation Index (ESCI) journals (74.3%) and 47th out of 78 SCIE (Science Citation Index Expanded) and ESCI journals (39.8%) [17]. To receive a higher JCI value, a higher citation frequency of citable articles will be essential. The 2021 JCI may be announced in June 2022 by Clarivate. I anticipate a higher 2021 JCI than that in 2020.

**Fig. 3.**
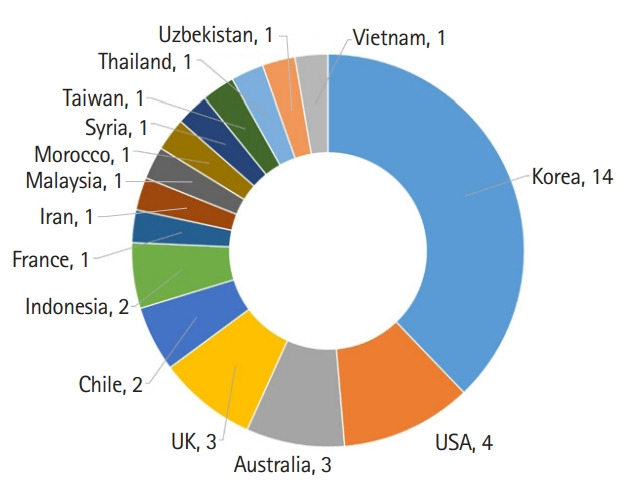
Number of articles in *Journal of Educational Evaluation for Health Professions* according to the authors’ country in 2021.

**Fig. 4.**
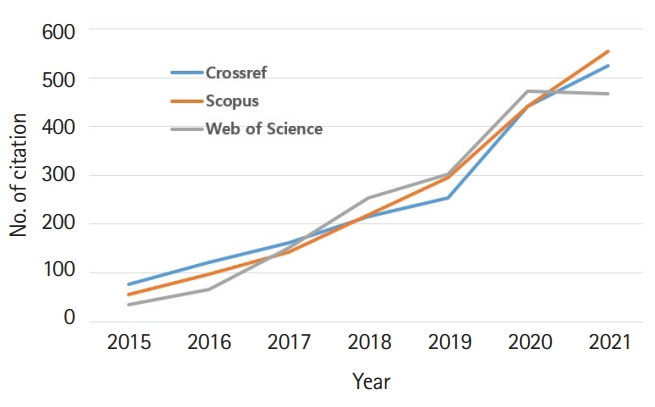
Total cites of *Journal of Educational Evaluation for Health Professions* in Crossref metadata, Scopus, and the Web of Science Core Collection (WOS) from 2015 to 2021.

Bibliometric statistics for the author’s country and total cites are presented in Figs. 3 and 4 (Supplement 2), respectively. There were authors from 15 countries, mainly from Asia, which reflects the regional scope of JEEHP. The number of total cites continuously increased. In 2021, JEEHP was cited 525 times in the Crossref metadata, 555 times in Scopus, and 461 times in Web of Science Core Collection. The manually calculated impact factor in Web of Science increased to 1.846 in 2021 from 1.254 in 2020. The CiteScore Tracker 2021 calculated by Scopus was 2.5 (366 citations 2018 to date/145 documents 2018 to data), last updated on January 5, 2022, which is available from: https://www.scopus.com/sourceid/21100467423. This value is higher than that in 2020 (1.7)

Table 1 presents journal statistics from 2021. The number of unsolicited submissions (n=296) increased from 2020 (n=275). However, the number of publications decreased from 44 in 2020 to 33 in 2021. This primarily originates from the low number of commissioned articles in 2021. The acceptance rate of unsolicited manuscripts (8.9%) was lower than that in 2002 (10.6%). In 2020, the first year of COVID-19, the number of submissions (n=286) was nearly double compared to 2019 (n=147). This sharp increase was a common phenomenon seen in other scholarly journals in Korea [18]. The amount of submissions to this journal was consistent in 2021.

I should be cautious when selecting manuscripts for review. The number of rejected or withdrawn manuscripts after review was 16 in 2020 and 10 in 2021. The number of accepted manuscripts after review was 26 out of 43 (70.3%) in 2021. In 2022, the editorial office will do its best to select acceptable manuscripts for review to save reviewers’ time.

## Appreciation to reviewers and volunteers

I am deeply indebted to the reviewers who voluntarily devoted themselves to reviewing manuscripts. Their role is essential for maintaining the journal quality. I regret that I sent them some manuscripts that were finally rejected. Below are the reviewers’ names and affiliations by country.

Australia: Boaz Shulruf, University of New South Wales; Elio Stefan Arruzza, University of South Australia
Chile: Castillo Niño Manuel, University of Chile
Indonesia: Armyanti Ita, Tanjungpura University; Romiko, Muhammadiyah University of Palembang
India: Upreet Dhaliwal, University of Delhi; Kiran Goswami, All India Institute of Medical Sciences; T. S. Gugapriya, All India Institute of Medical Science, Manjiri Phansalkar, Pondicherry Institute of Medical Sciences
Israel: Colin Block, The Hebrew University of Jerusalem
Italy: Colaceci Sofia, Saint Camillus International University of Health Sciences
Korea: A Ra Cho, The Catholic University of Korea; Yera Hur, Hallym University; Junyong In, Dongguk University; Hyun Kang, Chung-Ang University; Jae Hyun Kim, Dankook University; Mi Kyoung Yim, Korea Health Personnel Licensing Examination Institute; Sun Kim, The Catholic University of Korea; Young Ju Kim, Ewha Womans University; Dong Kyu Lee, Dongguk University; Keumho Lee, Korea Institute for Research in the Behavioral Sciences; Younjae Oh, Hallym University; Janghee Park, Soonchunhyang University; Jeong Yun Park, University of Ulsan; Jungchul Park, Dankook University; Jungkyu Park, Kyungpook National University; Kyung Hye Park, Yonsei University; Song Yi Park, Dong-A University; Won Kyun Park, Keimyung University; Dong Gi Seo, Hallym University; Ji-Hyun Seo, Gyeongsang National University; Aeree Son, Samyook University; Sanghee Yeo, Kyungpook National University; Hyun Bae Yoon, Seoul National University; Dong-Mi Yoo, The Catholic University of Korea
Malaysia: Fata Nahas Abdulrahman, International Islamic University; Jamshid Shazia, Sultan Zainal Abidin University
Morocco: Hassouni Kenza, Mohammed VI University of Health Sciences; El Hajji Mohamed, Ibn Zohr University
Pakistan: Rano Mal Piryani, Liaquat University of Medical and Health Sciences
Portugal: José Miguel Padilha, Oporto Nursing School
South Africa: Richard Cooke, University of the Witwatersrand
Thailand: Poonpong Boonbrahm, Walailak University; Marisa Krairiksh, Khon Kaen University; Manoch Namfu, Rajamangala University of Technology Lanna
United Kingdom: Oliver Kemp, Gloucestershire NHS Foundation Trust; Marios Nicolaides, Queen Mary University of London; Cesar A. Orsini, Norwich Medical School, University of East Anglia; Emily Suckling, University Hospitals Bristol and Weston NHS Foundation Trust
United States of America: Chad Cook, Duke University; Robert Cunningham, Maryville University; Mariana D’amico, Nova Southeastern University; Jessica Fino, Midwestern State University; Sandra Groth, Midwestern State University; Junguk Hur, University of North Dakota; Myunghee Jun, University of Wisconsin-Green Bay; Hyekyung Kim, University of Wisconsin-Green Bay; Seock-Ho Kim, The University of Georgia; Amteshwar Singh, Johns Hopkins University; Jessyca Wagner, Midwestern State University
Vietnam: Bach Xuan Tran, Ha Noi Medical University

Tom Huh, a graduate student in the Division of Life Sciences, College of Life Sciences and Biotechnology, Korea University, Seoul, Korea, voluntarily made audio recordings of some abstracts.

## Which journal policies will be emphasized in 2022

In the screening process of a submitted manuscript, the fitness of the manuscript in terms of both aims and scope and style and format will be meticulously checked before dispatching it for review. Reviewers or editors cannot determine originality; this determination is dependent on readers’ experience. Therefore, the review has been focused on scientific integrity. A description according to reporting guidelines will be more strictly required. It should be demonstrated that the study design follows specific reporting guidelines. JEEHP is a non-commercial scholarly journal, so the number of published articles is not critical—but scientific integrity is essential. The editorial team and I will do our best to maintain these policies in 2022 to save reviewers’ time.

## Figures and Tables

**Table 1.** Journal statistics of manuscripts submitted to Journal of Educational Evaluation for Health Professions from January 1 to December 31, 2021

	No. of manuscripts	Content
Manuscripts submitted	302	
No. of commissioned manuscripts	6	Editorial, 4; review, 2
No. of unsolicited manuscripts	296	
Manuscripts under re-submission, review, or revision	6	Under re-submission, 4; under review, 2
Manuscripts rejected without review	253	Unsuitable, 250; other reasons, 3
Manuscripts reviewed out of 290 unsolicited manuscripts	37	Accepted and published, 26; rejected 10; withdrawn, 1
No. of publications out of 296 submitted manuscripts in 2021	32	One article published in 2021 was submitted in 2020
No. of publications out of 290 unsolicited manuscripts	26	Opinion, 1; review, 4; research article, 14; brief report, 5; educational/faculty development material, 2
Acceptance rate overall (%)	10.8	32/296=0.108
Acceptance rate of unsolicited manuscripts (%)	8.9	26/290=0.089
Median time from submission to the first decision (day)	15	
Median time from submission to publication (day)	43	
Median time from acceptance to publication (day)	1	

## Supplementary materials

Supplement 1. Number of articles on the 4 types of the metaverse in PubMed by year with the search term “(((augmented reality) OR (lifelogging)) OR (mirror world)) OR (virtual reality)” [cited 2022 Jan 8], presented in Fig. 1.

Supplement 2. Total cites of *Journal of Educational Evaluation for Health Professions* in Crossref metadata, Scopus, and the Web of Science Core Collection (WOS) from 2015 to 2021, presented in Fig. 4.
